# TNFSF10: a promising prognostic biomarker and therapeutic target for immunotherapy in testicular germ cell tumors

**DOI:** 10.3389/fimmu.2026.1761075

**Published:** 2026-04-28

**Authors:** Guangmin Liu, Lin Du, Shanshan Lv, Zhizhong Liu, Jian Cao, Liqing Fan, Shusheng Zhang, Kongrong Xu, Lei Xue

**Affiliations:** 1 Department of Pathology, The Affiliated Cancer Hospital of Xiangya School of Medicine, Central South University/Hunan Cancer Hospital, Changsha, China; 2 National Health Commission Key Laboratory of Human Stem Cell and Reproductive Engineering, Institute of Reproductive and Stem Cell Engineering, Central South University, Changsha, China; 3 Department of Laboratory Medicine, Dongying People’s Hospital, Dongying, China; 4 Clinical Research Center for Reproduction and Genetics in Hunan Province, Reproductive and Genetic Hospital of CITIC-Xiangya, Changsha, China; 5 Department of Urology, the Affiliated Cancer Hospital of Xiangya School of Medicine, Central South University/Hunan Cancer Hospital, Changsha, China; 6 The First Hospital of Changsha, The Affiliated Changsha Hospital of Xiangya School of Medicine, Central South University, Changsha, China; 7 Reproductive Medicine Center, Maternal and Child Health Hospital of Guangxi Zhuang Autonomous Region, Nanning, China

**Keywords:** immune cell infiltration, immunotherapy, prognostic marker, testicular germ cell tumor, TNFSF10

## Abstract

**Background:**

Testicular germ cell tumors (TGCTs) are malignant neoplasms with a poor prognosis, and the absence of reliable biomarkers for patient stratification and diagnosis presents a significant challenge.

**Methods:**

We employed an integrated analysis of Single-Cell RNA-Sequencing and TCGA data to evaluate TNFSF10 as a potential biomarker for prognosis and immunotherapy in TGCTs.

**Results:**

Our findings revealed that aberrant TNFSF10 expression was significantly associated with patient survival outcomes. Silencing TNFSF10 inhibited the proliferation, migration, and invasion of TGCT cells, highlighting its role in tumor progression. Moreover, TNFSF10 expression was closely correlated with various components of the tumor microenvironment, suggesting its involvement in both the biological behavior of TGCTs and their response to treatment.

**Conclusion:**

TNFSF10 emerges as a promising diagnostic and prognostic biomarker for stratification and targeted therapy in TGCTs, offering new prospects for personalized treatment strategies.

## 1 Introduction

TGCTs are a broad category of tumors originating from the germinal epithelia of seminiferous tubules in the testes. The global incidence of TGCTs has gradually increased with an annual growth rate of 1%–2% since the 20th century, the number of diagnoses is also expected to increase in the future (1, 2). TGCTs are the most common solid malignancy among men aged 15–40 years old (3). Histologically, TGCTs are categorized as seminomas (50%–60% of tumors), non-seminomas (40%–50%), and spermatocytic tumors (<1%) (4, 5). Seminomas are typically diagnosed at around 35 years of age, and non-seminomas are diagnosed at around 25 years (4), with both forms arising from germ cell neoplasia *in situ* (GCNIS) (4, 5). Yao et al. revealed distinct differences in gene expression profiles, chromosomal instability patterns, and somatic mutation landscapes across different subtypes of testicular germ cell tumors TGCTs (6). The standard clinical treatment method for TGCTs is orchiectomy, supplemented with chemotherapy and radiation therapy (7). Currently, approximately 95% of patients with TGCTs are cured, which explains why the population of survivors is expanding (8). However, although patients are cured, they may face potential adverse effects and a poorer quality of life later in life. Survivors are reported to have twice the risk of recurrence of malignant tumors after chemotherapy and radiation, and there is evidence of a dose-dependent relationship (8). In addition, normal spermatogenesis and sexual function could be impaired in patient post-treatment, and, in some cases, this could lead to the development of secondary tumors and cardiovascular disease (7). Therefore, elucidating the pathogenesis and molecular characteristics of tumor development is necessary to identify effective noninvasive biomarkers and therapeutic targets for precision therapy.

Rapid technological advances have expanded our understanding of diseases at the cellular and molecular level. Single-cell RNA-sequencing (scRNA-Seq) has helped us determine gene expression profiles with unprecedented resolution. This has aided the quantitative analysis of gene expression at the single-cell level to reveal the correlations among heterogeneity, signaling pathways, drug resistance, and tumor microenvironment. Mo et al. performed an scRNA-Seq study on testicular seminomas and reported the gene expression characteristics of testicular seminoma cells at the single-cell level (9). We identified eleven potential predictors for TGCTs by analyzing the data reported by Mo et al. and found that TGCTs are associated with poor prognosis, according to the expression of the eleven genes. The expression of Tumor Necrosis Factor Superfamily Member 10 (TNFSF10), one of these genes, was associated with survival. TNFSF10, encoding TRAIL, is a multifunctional TNF superfamily member (10). Beyond its canonical pro-apoptotic role, TRAIL regulates immune homeostasis, induces immunogenic cell death (ICD), and promotes inflammation via NF-κB and NLRP3 inflammasome activation. It also amplifies endoplasmic reticulum stress, critically contributing to the pathogenesis of autoimmune and inflammatory diseases (11). The role of TNFSF10 in tumors has been reported. Oh and Sun showed that TNFSF10 plays a role in regulating cancer invasion and metastasis (12). Jiang et al. confirmed that TNFSF10 expression could promote cancer cell apoptosis in hepatocellular carcinoma (13). In melanoma, TNFSF10 expression contributed to anti-tumor activities (14). However, the function of TNFSF10 in TGCTs remains unclear.

In this study, we systematically analyzed the expression of marker genes in TGCT tumor cells. Our findings showed that aberrant TNFSF10 expression was significantly correlated with the survival of patients with TGCT and silencing of TNFSF10 inhibited the proliferation, migration and invasion of TGCTs cells. In addition, TNFSF10 expression was strongly correlated with the expression of TME components. Thus, it serves as a potential diagnostic and prognostic biomarker for stratification and drug therapy in patients with TGCTs.

## 2 Materials and methods

### 2.1 Data source and preprocessing

The testicular seminoma scRNA-Seq dataset GSE197778 was retrieved from the GEO database (9). The percentage of mitochondria and rRNA was calculated using the Percentage Feature Set function, and the number of genes expressed in each cell were greater than 500 and less than 8000. The mitochondrial content was less than 30%. In addition, the number of unique molecular identifiers (UMI) in each cell was at least 500. Public clinical data and gene expression information were retrieved from the TCGA database (https://portal.gdc.cancer.gov/). In all, data from 134 samples in the TCGA-TGCT cohorts were used for further analysis.

### 2.2 Cell clustering analysis

We embedded and clustered scRNA-Seq data using Uniform Manifold Approximation and Projection (UMAP), a manifold learning approach for dimensionality reduction, which can preserve the global data structure better than other similar techniques (15). This technique is included in the Seurat package; the settings were set to default. The resultant cluster map was annotated according to the cell types.

### 2.3 Risk factor and survival analysis

**Figure 1:**
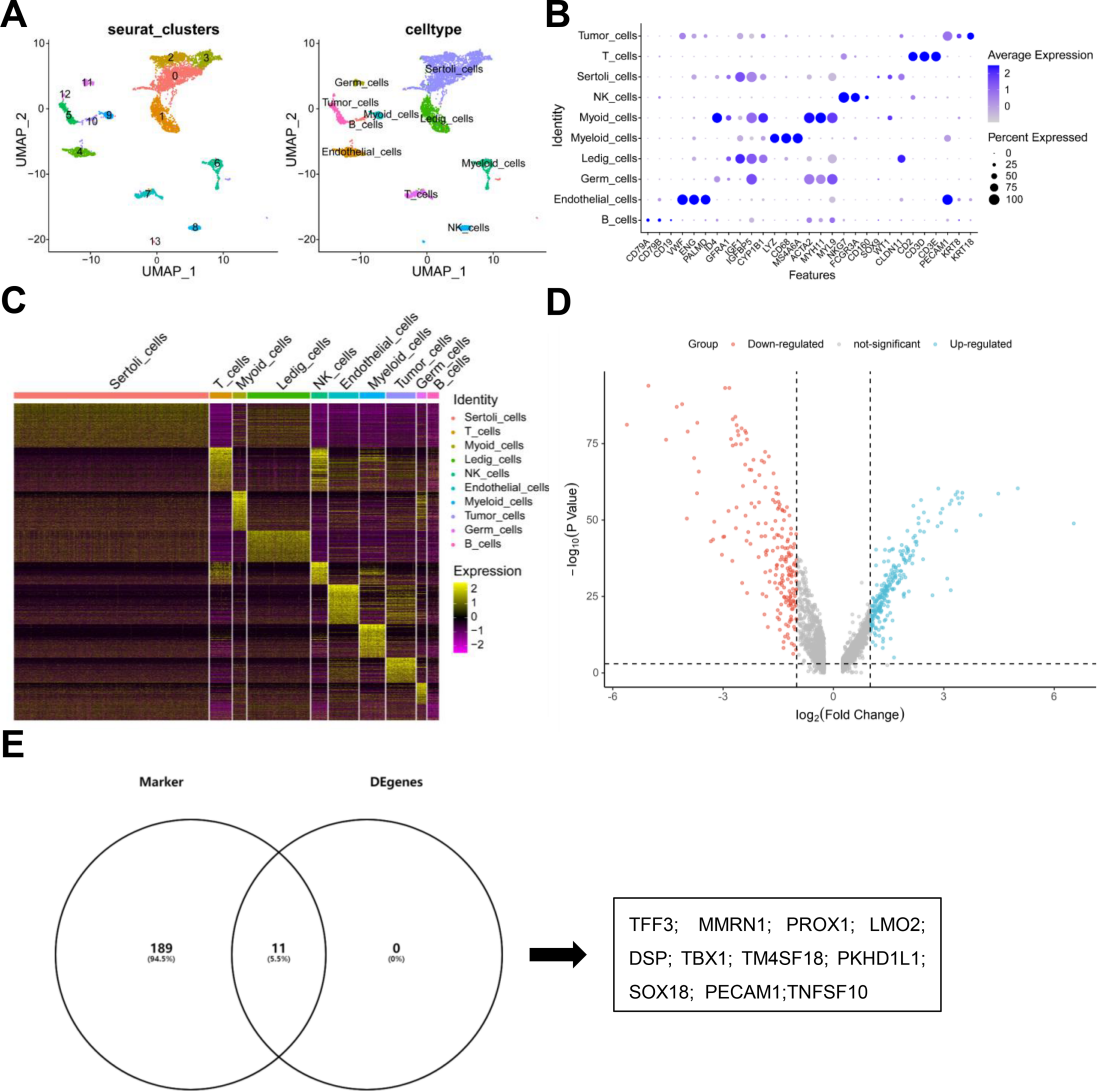
Single-cell gene expression programs define the tumor marker genes in TGCTs. **(A)** Cells were clustered in two dimensions using the UMAP dimensionality reduction technique and annotated using cluster labels and cell types. **(B)** A bubble chart showing the proportion of marker genes expressed and the average expression levels in different cell types. **(C)** Heatmap showing the top 200 genes with high expression levels in different cell types. **(D)** A volcano map of differentially expressed genes between tumor cells and germ cells. **(E)** Venn diagram of tumorigenesis-related genes.

Using the Cox proportional hazards regression model, risk scores were calculated for each sample based on the Z-score-normalized expression values of the 11 genes selected in **Figure 1E**. Samples were then dichotomized into high-risk and low-risk groups according to the median risk score. To construct the prognostic model, a univariate Cox regression model was used to analyze the correlation between gene expression and survival outcomes using ASSISTANT for Clinical Bioinformatics (https://www.aclbi.com/static/index.html#/). The correlation between TNFSF10 expression and survival was calculated using BEST (https://rookieutopia.hiplot.com.cn/app_direct/BEST/) (16).

### 2.4 TNFSF10 expression and copy number alteration analysis

RNA sequencing data in the TPM format for TNFSF10 expression in normal and tumor tissues were recorded from TCGA-TGCTs and GTEx datasets, and the results were analyzed using GEPIA2 (http://gepia2.cancer-pku.cn/#index) (17). Histograms of TNFSF10 mutation, CNA frequency, and distant metastasis in TGCTs were analyzed using cBioPortal (https://www.cbioportal.org/ (18).

### 2.5 Functional enrichment analysis in TGCTs

TNFSF10 association enrichment analysis was performed using BEST (https://rookieutopia.hiplot.com.cn/app_direct/BEST/). Gene Ontology (GO) enrichment analysis, Kyoto Encyclopedia of Genes and Genomes (KEGG) analysis, and Gene Set Enrichment Analysis (GSEA) were conducted to detect phenotypes, biological process, and signaling pathways.

### 2.6 The TME and immune checkpoint blockade therapy analysis

The correlation between TNFSF10 expression and the expression of major histocompatibility complex (MHC) molecules, immunomodulators, key immune checkpoint proteins and drugs from the TCGA-TCGT data were analyzed using BEST (https://rookieutopia.hiplot.com.cn/app_direct/BEST/). Tumor Immune Dysfunction and Exclusion (TIDE) is a comprehensive score indicating immune escape and tumor immune dysfunction; this considers tumor-infiltrating cytotoxic T lymphocyte (CTL) dysfunction and rejection by immune checkpoints. RNA-Seq raw count data and corresponding clinical information from TCGA-TCGT data were evaluated by the TIDE algorithm to predict the potential immune checkpoint blockade (ICB) response (19). We conducted our analysis using ASSISTANT for Clinical Bioinformatics (https://www.aclbi.com/static/index.html#/).

### 2.7 Immunohistochemical staining

Paraffinized sections of human testicular tumor were deparaffinized in xylene and rehydrated. The slides were blocked and treated overnight with anti-TNFSF10 (ABclonal, A2138, China) antibodies at 4 °C. The next day, the slides were treated with HRP-IgG secondary antibodies, followed by protein detection using DAB solution. Images were acquired using a microscope (Olympus, KF-PRO-005).

### 2.8 TGCT cell culture and siRNA transfection

TCAM-2, a human TGCT cell line, was obtained from Dr. Yuxin Tang (20, 21). TCAM-2 cells were cultured in Dulbecco’s Modified Eagle’s Medium (DMEM, GIBCO, USA) containing 10% fetal bovine serum (FBS, GIBCO, USA), 100 U/ml penicillin and 100 mg/ml streptomycin (GIBCO) and were incubated at 37°C under 5% CO2. SiRNAs targeting TNFSF10 were designed and synthesized by Tsingke Biotechnology Co., Ltd (Beijing, China). SiRNAs transfection were conducted using Lipofectamine 3000 transfection reagent (Invitrogen, Waltham, MA, USA) according to the manufacturer’s instructions. TNFSF10 siRNA1: CAACUCCGUCAGCUCGUUA UAACGAGCUGACGGAGUUG; TNFSF10 siRNA2: CAGAUGCAGGACAAGUACU AGUACUUGUCCUGCAUCUG; TNFSF10 siRNA-3: GCUGUAACUUACGUGUACUUU AAAGUACACGUAAGUUACAGC. The knockdown efficiency of the siRNAs was assessed by quantitative real-time reverse transcription PCR (qRT-PCR).

### 2.9 CCK-8 assay

The cell proliferation was detected by cell counting kit-8 (CCK--8) reagent (Invitrogen). TCAM-2 cells were seeded in a 96-well plate with 6 × 103 cells/mL (200μL/well) for 6, 24, 48, 72, 96, and 120h as day0, day 1, day2, day3, day4, day5, respectively. Then 20μL CCK-8 was added and incubated for 2h at 37 °C. The absorbance at 492 nm was determined by the enzyme immunoassay analyzer (Thermo Fisher Scientific, Waltham, MA, USA).

### 2.10 Plate colony formation assay

TCAM-2 cells were seeded in a 6-well plate (400 cells/well) and incubated at 37°C under 5% CO2 for about 10 days when most single-cell colonies consist of >50 cells. The cells were fixed in 4% paraformaldehyde and stained with 0.5% crystal violet. The colony number was counted.

### 2.11 Cell migration and invasion assays

The migration and invasion assays were conducted using 8.0μm Transwell Permeable Support (353097) (Corning Inc., Corning, NY, USA). 5 × 104 cells were seeded into the upper chamber in 200μl serum-free medium and 800μl medium containing 15% FBS was added to the lower chamber for 36h. Matrigel Matrix (BD Biosciences, San Jose, CA, USA) was added in the upper chamber in invasion assay. Next, cells were fixed in 4% paraformaldehyde and stained with 0.5% crystal violet and invaded cells was counted in six randomly selected fields.

### 2.12 Statistical analysis

Data were statistically analyzed using GraphPad Prism 7 or other related tools available online. Differences between two groups were calculated using a Student’s t-test. Survival analyses were conducted using the log-rank test. Correlation analysis was conducted using the Cox model. *P* values < 0.05 were considered statistically significant.

## 3 Results

### 3.1 Single-cell gene expression programs define tumor marker genes in TGCTs

A single-cell map of testicular seminoma with lymph node metastasis was constructed using scRNA-Seq. The characteristics of gene expression in testicular seminoma were determined using this method (9). We used the scRNA-Seq data obtained from the Gene Expression Omnibus accession GSE197778 for thorough analysis, we identified thirteen distinct cell clusters (**Figure 1A**, left). Cells from each cell type exhibited strong geometric separation, suggesting the validity of the clusters obtained. Testicular seminomas could be classified into ten different cell types, namely, tumor cells, T cells, Sertoli cells, NK cells, myoid cells, myeloid cells, Leydig cells, germ cells, endothelial cells, and B cells (**Figure 1A**, right). Additionally, we showed the marker genes for each cell type using bubble maps and defined the cell types based on their marker genes. These marker genes were identified from previous studies (4, 22). Obvious differences in gene expression were observed in each cell type. For example, tumor cells showed particularly high expression levels of VWF, KRT8, and KRT18, whereas IGF1 and MYH11 showed high expression levels in germ cells (**Figure 1B**). These results indicated that specially expressed marker genes may be used to identify the subgroups of cells in future studies. Based on the classification of the cell types, we identified the top 200 high-expression genes in every cell type (**Figure 1C**). We identified 438 differentially expressed genes in the tumor cell versus germ cell subset (233 upregulated and 205 downregulated) (**Figure 1D**). For further investigation, we selected eleven genes, which were not only expressed in the top 200 tumor cells but were also differentially expressed in tumor cells versus germ cells (**Figure 1E**). These genes were TFF3, MMRN1, PROX1, LMO2, DSP, TBX1, TM4SF18, PKHD1L1, SOX18, PECAM1, and TNFSF10.

### 3.2 Evaluation of the clinical outcomes of TGCTs

**Figure 2:**
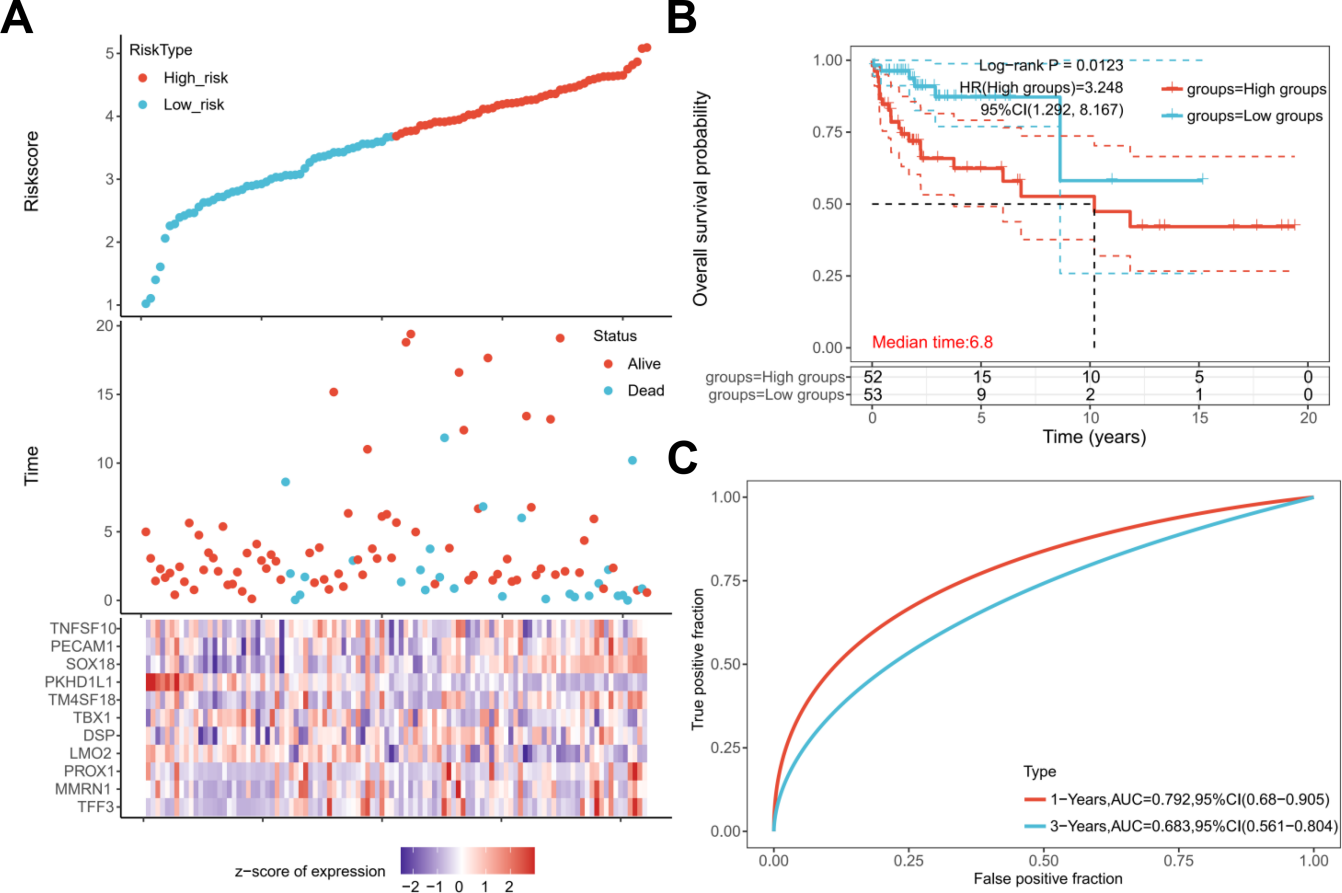
Development of the prognostic index based on the expression of tumor-related genes. **(A)** Risk score curve showing the survival of patients and expression profiles of the eleven prognostic genes in low- and high-risk groups. **(B)** Kaplan-Meier curve showing the eleven genes for which gene expression was negatively associated with the clinical survival of patients, based on data from the TCGA-TGCT database. The median survival duration (years) corresponds to a 50% survival rate. **(C)** The ROC curve and AUC of the risk model at different times.

**Figure 3:**
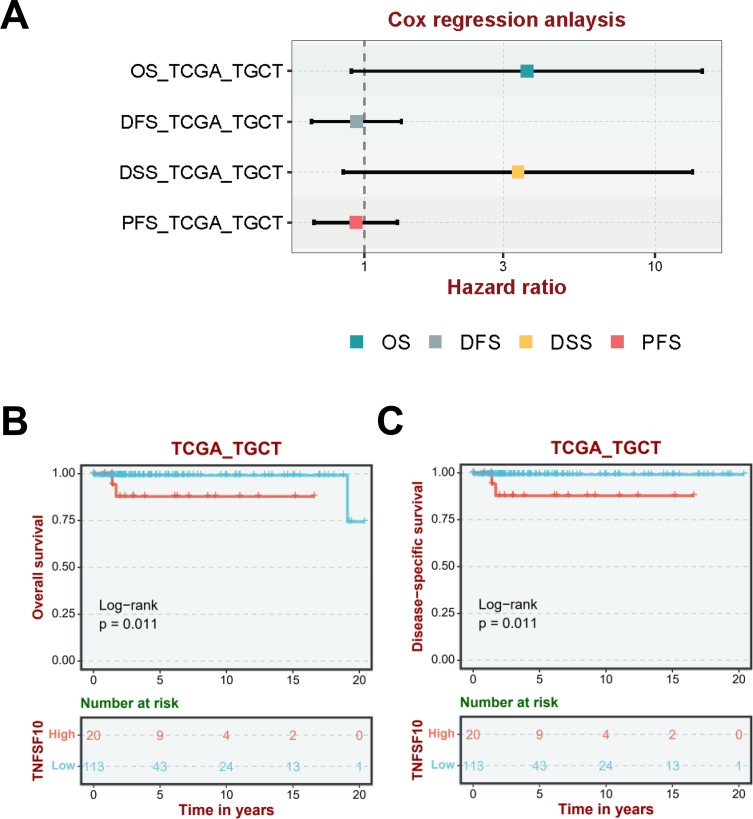
Association of TNFSF10 expression with survivals in patients with TGCT. **(A)** Analysis and Visualization of Hazard Ratios derived from Cox Regression Analysis. Kaplan-Meier survival curves showing the results of log-rank tests for the OS **(B)**, DSS **(C)** of patients with TGCT.

To detect the clinical value of the eleven genes at the mRNA level in TGCTs, we gained the raw RNA-Seq data (Level 3) from 134 cases and the corresponding clinical information from the TCGA dataset (https://www.cancer.gov/). Accordingly, patients were divided into two groups: the high-risk group and low-risk group (**Figure 2A**, top). The elevated expression of the eleven genes was relevant to elevated mortality rates (**Figure 2A**, middle) and a higher risk score (**Figure 2A**, bottom). The findings from Kaplan-Meier (K-M) survival analysis also confirmed that a high expression of the eleven genes indicated poor prognosis (log-rank p=0.0123, HR = 3.248) (**Figure 2B**). And, the area under ROC curve of the genes set was greater than 0.5, indicating that it had good predictive power (**Figure 2C**). In addition, elevated TNFSF10 expression served as an independent prognostic factor for inferior overall survival (OS) and disease-specific survival (DSS) in patients with TGCTs, with multivariate Cox regression analysis yielding hazard ratios (HR) exceeding 3.0 for both endpoints (HR > 3.0, P < 0.05). In contrast, TNFSF10 expression levels showed no statistically significant association with disease-free survival (DFS) or progression-free survival (PFS) in the same analytical framework(**Figure 3A**). The number of death cases is 4. K-M curve analysis revealed that low TNFSF10 expression is a better prognostic predictor for OS in TGCTs (**Figure 3B**). Meanwhile, low TNFSF10 expression was associated with prolonged DSS in TGCTs (**Figure 3C**). Furthermore, our analysis revealed that TNFSF10 exhibited the strongest correlation with adverse prognosis in patients with testicular germ cell tumors (TGCTs). These results showed that TNFSF10 could be a prognostic marker for TGCTs. Consequently, TNFSF10 was selected as a candidate molecular target for subsequent functional investigation.

### 3.3 Gene expression and CNA analysis of TNFSF10

**Figure 4:**
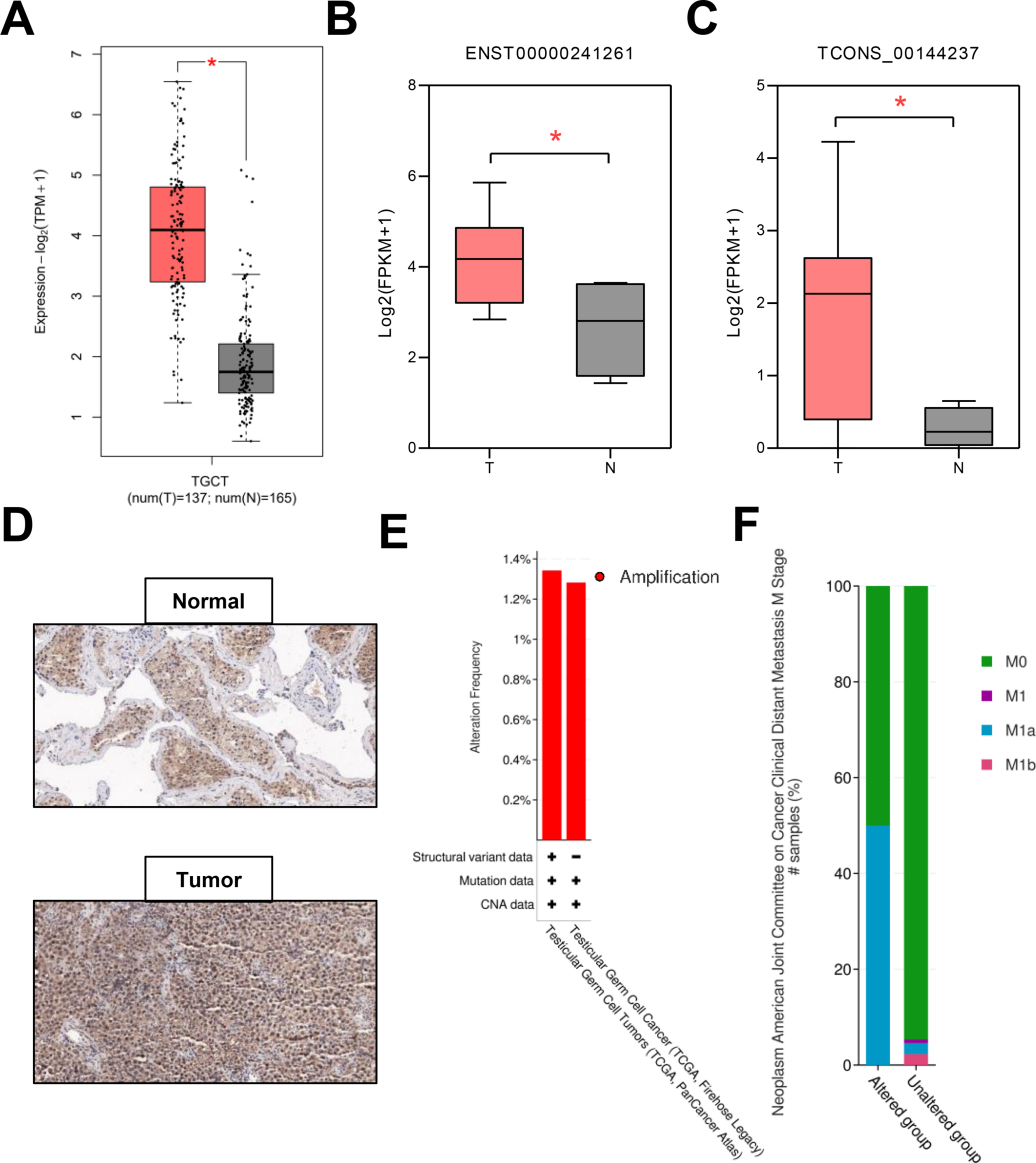
Gene expression and mutation analysis of TNFSF10. **(A)** TNFSF10 is highly expressed in TGCTs. **(B-C)** Two transcripts of TNFSF10 were expressed at high levels in our TGCTs RNA-Seq data. **(D)** Immunohistological staining showed that TNFSF10 was expressed at high levels in TGCTs. **(E)** Histogram of TNFSF10 expression alteration frequency with amplification in TGCTs derived using cBioPortal. **(F)** Distant metastasis of TGCT associated with alterations in the TNFSF10 gene. *p value < 0.01.

TNFSF10 expression patterns in human TGCT tissues and adjacent normal tissues were determined from GEPIA2 datasets. TNFSF10 expression was significantly elevated in TGCTs compared to that in adjacent normal testicular tissues (**Figure 4A**). Simultaneously, using published RNA-Seq data, we confirmed that two transcripts of TNFSF10 were expressed at higher levels in TGCTs than in adjacent normal testicular tissues (23) (**Figures 4B, C**). In addition, Pan-Cancer Analysis of TNFSF10 expression patterns were different in human different tumors and adjacent normal tissues, which determined from GEPIA2 datasets (**Supplementary Figure 1**). Immunohistochemical (IHC) staining showed that anti-TNFSF10 expression was higher in TGCTs than in the control group (**Figure 4D**). To explore the cause underlying TNFSF10 dysregulation, we comprehensively analyzed the factors associated with TNFSF10 expression. We performed a comparative analysis of TNFSF10 expression to determine the CNA status of TNFSF10 in TGCTs using the cBioPortal database. The CNA profiles showed that amplification was an important factor in TNFSF10 expression. Gene alterations were identified in 1.34% of 149 cases from TCGA-TGCT (PanCancer Atlas) and in 1.28% of 156 cases from TCGA-TGCT (Firehose Legacy) (**Figure 4E**). This suggested that frequent copy number alterations in TNFSF10 were correlated with elevated TNFSF10 expression. Tumor metastasis, the progressive movement of tumor cells from the primary site to distant organs, is the major cause of cancer-related deaths (24–26). We found that genetic alterations in TNFSF10 are more important in distant metastasis (**Figure 4F**). Therefore, genetic alterations could contribute to TNFSF10 dysregulation.

### 3.4 Silencing of TNFSF10 inhibited the proliferation, migration and invasion of TGCT cells

**Figure 5:**
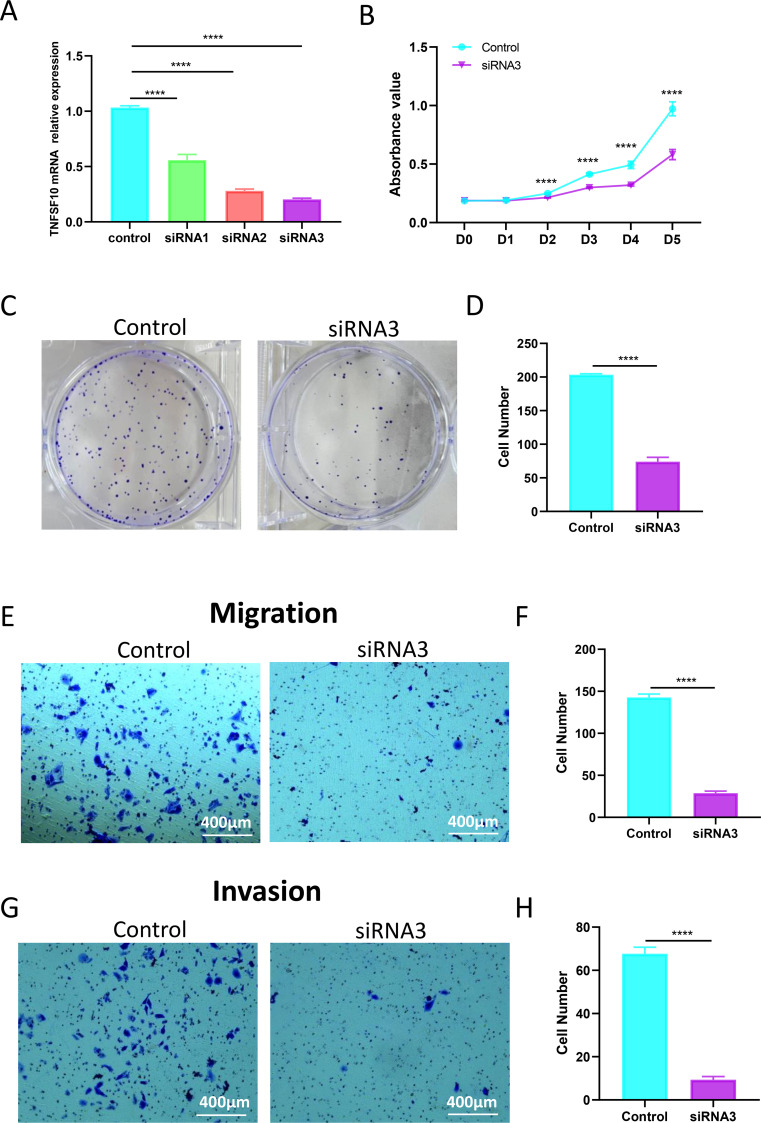
Silencing of TNFSF10 inhibited the proliferation, migration and invasion of TGCT cells. **(A)** The efficiency of TNFSF10 siRNAs on the TGCT cell line was assessed by qRT-PCR. **(B)** Cell proliferation was evaluated in CCK-8 assays. **(C)** Colony formation ability was evaluated in colony formation assays. **(D)** Cell colony formation rate. **(E)** Transwell cell migration assays showing migration of TGCT cells silencing TNFSF10. **(F)** The relative number of migrated cells in the control and TNFSF10 groups. **(G)** Transwell cell migration assays showing invasion of TGCT cells silencing TNFSF10. **(H)** The relative number of invasion cells in the control and TNFSF10 groups. siRNA: small interfering RNA; **** P < 0.0001.

To validate the biological functions of TNFSF10 in TGCTs, we selected TCAM-2 for *in vitro* experiments. The expression levels of TNFSF10 in TCAM-2 was determined using qRT-PCR after added TNFSF10 siRNA (**Figure 5A**). The proliferation was evaluated by the CCK-8 assay and clonal formation. The proliferation of TCAM-2 cells was inhibited by transfecting TNFSF10 siRNA in CCK-8 assay (**Figure 5B**). And silencing TNFSF10 inhibited the colony formation ability of TCAM-2 cells (**Figures 5C, D**). Transwell assay was used to investigated the migration and invasion abilities of TGCT cells (**Figures 5E–H**). Silencing TNFSF10 decreased the number of migrated and invaded cells significantly. This indicates that TNFSF10 plays an important role in the migration and invasion of TGCT, which warrants further investigation. These results indicated that silencing of TNFSF10 inhibited the proliferation, migration and invasion of TGCT cells.

### 3.5 Functional enrichment indicated that TNFSF10 expression is potentially associated with the TME in TGCTs

**Figure 6:**
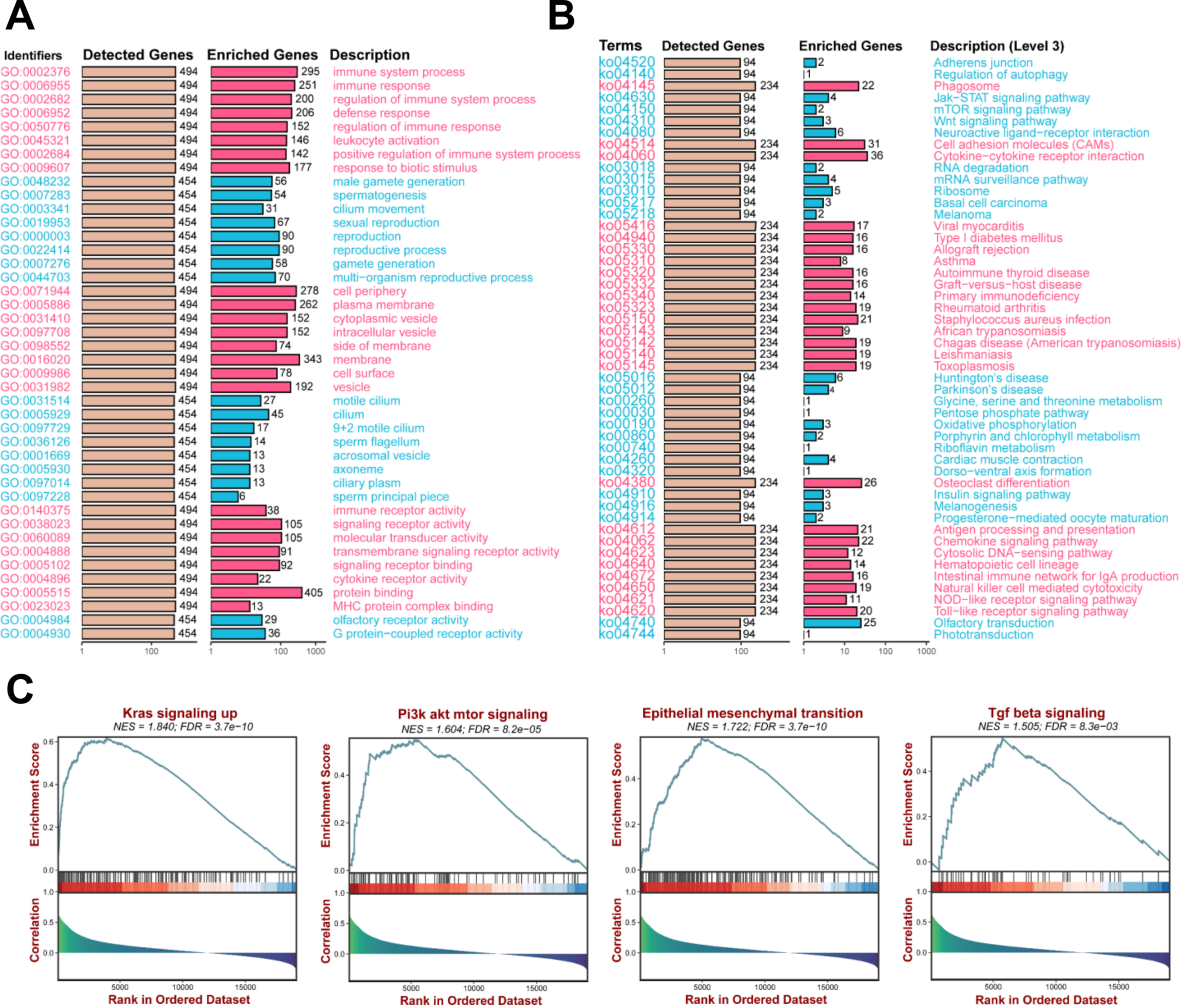
TNFSF10 expression is potentially associated with the expression of tumor microenvironment components in TGCTs. **(A, B)** GO functional and KEGG pathway analyses of TNFSF10. **(C)** GSEA for the regulatory signaling pathways involving TNFSF10.

Functional enrichment analyses were performed to investigate the functional mechanisms of TNFSF10. GO enrichment analysis was used to evaluate the potential mechanism of action of TNFSF10 considering the molecular function, biological process, and cellular component categories. TNFSF10 was functionally associated with several immune-related biological processes, including immune system processes, immune responses, and regulation of immune processes (**Figure 6A**). KEGG pathway analysis showed that TNFSF10 is involved in phagosome function, cell adhesion molecule production, and cytokine-cytokine receptor interaction signaling pathways (**Figure 6B**). GSEA was also used to identify the TNFSF10-associated pathways. GSEA results showed that TNFSF10 expression was associated with the activation of immune pathways, including the KRAS signaling pathway, PI3K-AKT signaling pathway, epithelial-mesenchymal transition, and TGF-β signaling pathway (**Figure 6C**). These findings implied that TNFSF10 may play a role in the TME.

### 3.6 TNFSF10 expression is associated with immune cell infiltration

**Figure 7:**
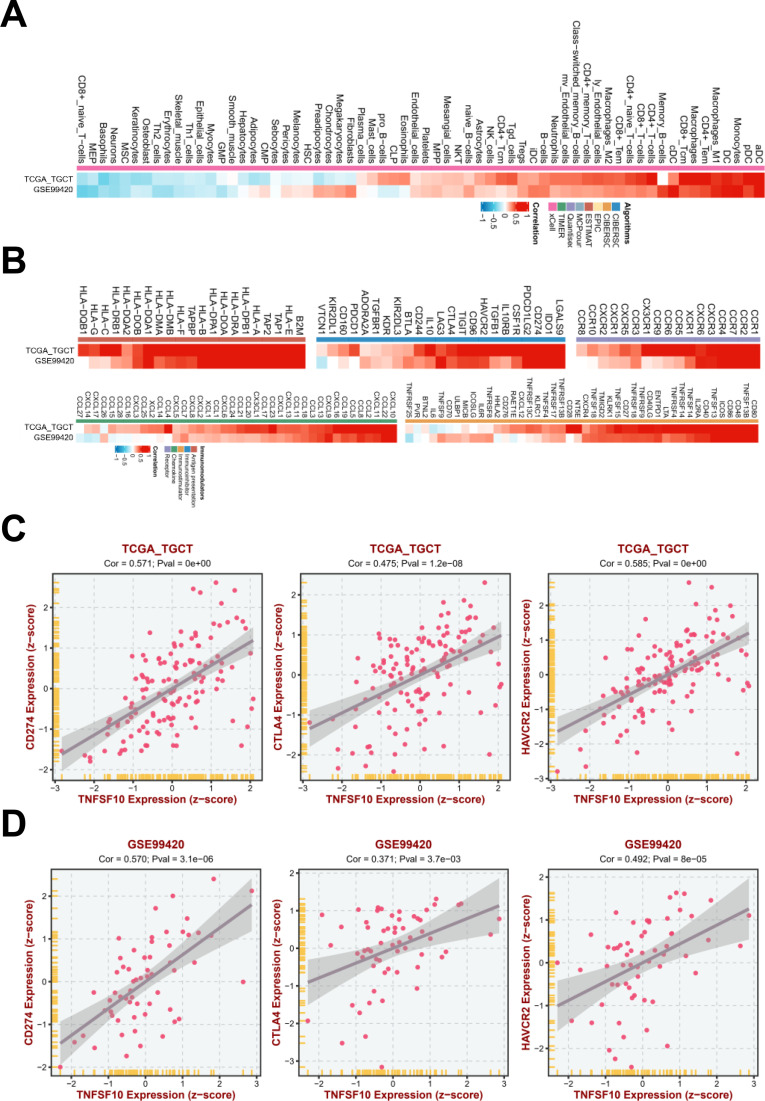
TNFSF10 expression showed a positive correlation with the key immune genes. **(A)** TNFSF10 expression was correlated with the infiltration of key immune cell types. **(B)** TNFSF10 expression was correlated with the expression of immunomodulators. **(C, D)** TNFSF10 expression showed a significant positive correlation with the expression of the key immune genes CD274, CTLA4, and HAVCR2 in the TGCA-TGCTs and GSE99420 database.

The results of functional enrichment analyses also indicated that TNFSF10 may regulate the TME by influencing the immune response. The TME is the cellular environment in which tumor cells are present. It comprises an extracellular matrix, soluble molecules, and tumor stromal cells. In the TME, immune cells and stromal cells, two primary types of non-tumor components, have potential value in the clinical diagnosis and prognostic evaluation of tumors (27). The TME has gained prominence in cancer immunity research. Immune cell infiltration plays a vital role in anti-tumor immunotherapy. Therefore, we analyzed the correlation between TNFSF10 expression and the abundance of key immune cell types using BEST. TNFSF10 expression was positively correlated with the infiltration of immune cells, such as dendritic cells, macrophages, CD4+ T cells, and CD8+ T cells (**Figure 7A**). Thus, TNFSF10 expression comprehensively regulates the TME via immune cell infiltration.

To further explore the regulatory mechanisms underlying TNFSF10 expression-related tumor immune infiltration, we further analyzed the correlation between TNFSF10 expression and the levels of immunomodulators, including antigen-presenting molecules, immunoinhibitors, immunostimulators, chemokines, and receptors, using TCGA datasets. The results indicated that TNFSF10 expression was highly correlated with the level of immunomodulators (**Figure 7B**). Notably, TNFSF10 expression showed a significant correlation with the expression of CD274 (PD-L1), CTLA4, and HAVCR2 (**Figures 7C, D**), which are therapeutic targets and predictive biomarkers used in cancer immunotherapy. For treatment with immune checkpoint inhibitors, CD274 (PD-L1), CTLA4, and HAVCR2 are known immune checkpoint proteins responsible for tumor immune escape. We combined these findings on immune checkpoint proteins and those obtained from subsequent analysis using the TIMER2 portal and further confirmed that TNFSF10 expression was positively correlated with immune checkpoint expression in TGCTs.

### 3.7 Correlation between TNFSF10 expression and the predicted outcomes of immunotherapy

**Figure 8:**
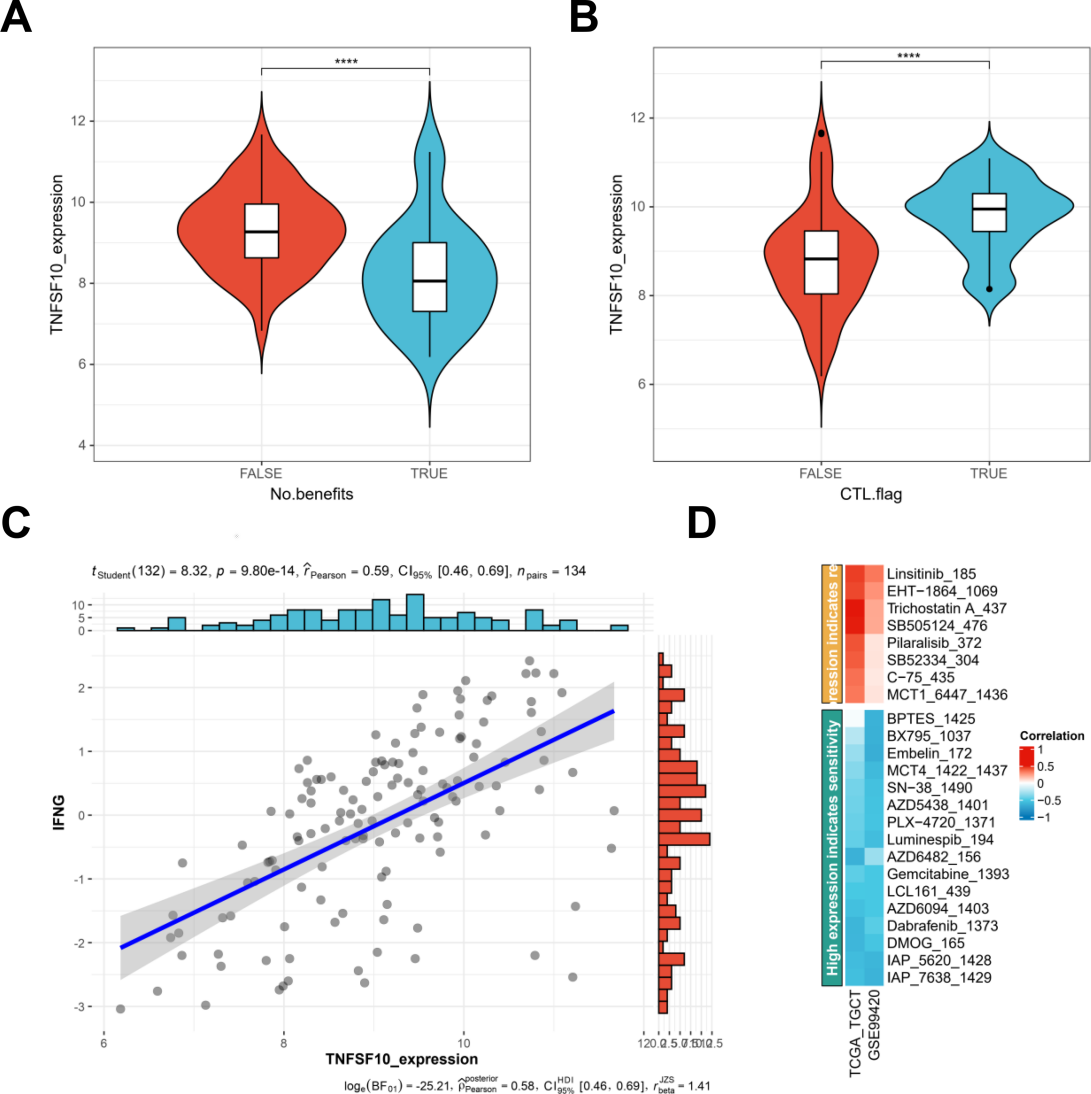
Correlation between TNFSF10 expression and the outcomes of immune therapy. **(A)** TNFSF10 expression showed a correlation with immune therapy using TIDE. **(B)** TNFSF10 expression showed a correlation with CTL.flag. **(C)** TNFSF10 expression showed a positive correlation with IFNG. **(D)** The correlation between the effects of each drug and TNFSF10 expression.

Tumor immunotherapy is a treatment in which tumor growth is controlled and tumor cells are eliminated through resuming regular anticancer immune responses and reactivating and sustaining the tumor immune cycles. These therapies include ICB and cell therapy. The effectiveness of ICB therapy depends on immune cell infiltration as well as immune checkpoints. The closed correlation between TNFSF10 and immune checkpoint expression implied that patients with TNFSF10 expression-associated tumors may respond well to immunotherapy. Therefore, the TIDE algorithm could be used to predict the therapeutic effect of ICB from the TCGA database. People with a high expression of TNFSF10 benefited from immune therapy (**Figure 8A**). TNFSF10 expression was correlated with the abundance of cytotoxic T lymphocytes (**Figure 8B**). TNFSF10 expression had a good correlation with the expression of the cytokine interferon-gamma (IFNG) (**Figure 8C**). The well-known inhibitor of anti-tumor immune activity, PD-L1, is induced to express itself by IFNG, which is essential for anti-tumor responses (28). We calculated the correlation between all drugs and TNFSF10 using BEST; our findings revealed more important drugs related to TNFSF10 expression (**Figure 8D**). The higher-ranked drugs indicated that a high level of TNFSF10 predicts drug resistance and vice versa.

## 4 Discussion

TGCTs are a broad category of malignant solid tumors occurring in men aged 15–40 years old (3). At present, it has a curative rate of>90% (4, 8). However, even though the men are cured, they face a two-fold greater risk of developing malignant neoplasms with recurrent metastases after treatment (8). In this study, we addressed the lack of diagnostic markers for men with tumor recurrence and metastasis. Exploring the molecular pathogenesis of TGCTs is crucial to fully understanding the development and prognosis of TGCTs. Mo et al. performed an scRNA-Seq investigation on a patient with a testicular seminoma diagnosis, identifying the features of tumor cells’ gene expression (9). Based on data analysis, we categorized testicular seminoma cells into ten cell types using different cell markers and identified eleven potential predictors for TGCTs prognosis that are only expressed in tumor cells. We found that the poor prognosis and survival of TGCTs were suggested by the high expression levels of the eleven genes. K-M curve analysis showed that low TNFSF10 expression is a better prognostic predictor for OS in TGCTs, indicating that TNFSF10 may be a biomarker for TGCTs. Furthermore, in our present study, we identified the molecular expression patterns and genetic alterations in TNFSF10 and investigated the correlation of TNFSF10 expression with immune cell infiltration and immune checkpoint gene expression.

According to data from the National Center for Biotechnology Information (NCBI), TNFSF10 is a cytokine classified within the tumor necrosis factor (TNF) ligand family. TNFSF10 preferentially induces apoptosis in transformed and tumor cells but does not appear to kill normal cells, even though it is expressed at significantly high levels in most normal tissues. TNFSF10 overexpression may be correlated with the high gene copy numbers. Genetic alterations could contribute to TNFSF10 dysregulation, which may influence the distant metastasis of tumor cells. The potential diagnostic and prognostic value of TNFSF10 and the molecular mechanisms underlying its effects in the TME in TGCTs remain unexplored. Previous studies have shown that TNFSF10 played significant roles in various cancers, including triple-negative breast cancer (29), esophageal squamous cell carcinoma (30), and hepatocellular carcinoma (13). These studies showed that TNFSF10 served as a promising biomarker for immune therapy in different cancers, but the role of TNFSF10 in TGCTs remains unclear. In this study, we first valeted that silencing of TNFSF10 inhibited the proliferation, migration and invasion of TGCT cells. TNFSF10 exerts context-dependent biological functions shaped by cellular identity, tumor microenvironment, and signaling milieu, manifesting dual pro-apoptotic and pro-tumorigenic activities. Therapeutic optimization thus requires precision strategies beyond pan-ligand inhibition—specifically, receptor subtype-selective targeting, modulation of downstream effectors, or rational combinatorial sensitization. Integration of biomarker-guided patient stratification with advanced delivery platforms (e.g., nanodelivery systems, engineered cellular therapies) further enhances targeting fidelity. This paradigm preserves TNFSF10-mediated tumor suppression while mitigating context-driven oncogenic risks, advancing toward safer, more effective clinical translation.

Tumor metastasis is a process of malignant tumor cells from the primary site to other sites to continue to grow through the lymphatic tract, blood vessels or body cavity. The metastasis of TGCTs is often the main reason for the failure of clinical treatment. We found that TNFSF10 could promote TGCT cells migration and invasion, which further proved that TNFSF10 can promote TGCTs development. The current findings require further validation across additional germ cell tumor-derived cell lines and *in vivo* animal models. The mechanism of TNFSF10 dysregulation in TGCT requires further research, although our results showed that TNFSF10 promote the migration and invasion abilities of TGCT cell lines. To our knowledge, our study is the first to characterize the functional role of TNFSF10 in TGCTs.

Functional enrichment analyses showed that TNFSF10 expression is functionally associated with several immunity-related biological processes and immune pathways, for example, the KRAS signaling pathway, PI3K-AKT signaling pathway, epithelial-mesenchymal transition, and TGF-β signaling pathway. These findings implied that TNFSF10 may play a role in the TME. The TME comprises all non-cancerous host cells present in the tumor, including fibroblasts, endothelial cells, neurons, adipocytes, adaptive cells, and innate immune cells; non-cellular components such as the extracellular matrix (ECM); and soluble products such as chemokines, cytokines, growth factors, and extracellular vesicles (27). The TME is becoming a focal point in cancer immunity research. In previous studies, immunotherapy for TGCTs was not investigated extensively (31). Schepisi et al. discuss the potential therapeutic strategies of immune checkpoint inhibitors and chimeric antigen receptor T-cell (CAR-T) therapy in testicular germ cell tumors (32). The role of TNFSF10 in TGCT immunotherapy needs to be explored. Our findings showed that TNFSF10 expression is positively correlated with the infiltration of immune cells, such as macrophages, CD4+ T cells, and CD8+ T cells. In addition, TNFSF10 expression is positively correlated with the expression of CD274 (PD-L1), CTLA-4, and HAVCR2, which are all immune checkpoints responsible for tumor immune escape. And we have demonstrated the expression pattern and function of TNFSF10 by experiments. These findings suggest that TNFSF10 emerges as a promising predictive biomarker for immunotherapy response by multi-scale transcriptomic comprehensive analysis. This is the first time found that TNFSF10 was correlated with immune response in TGCT. However, owing to the rarity of TGCT and the consequent difficulty in procuring an adequate number of clinical specimens, functional validation of these pathways at the clinical sample level was not feasible. And the immunomodulatory mechanism of TNFSF10 in TGCTs was not clear, which needs further research.

## 5 Conclusions

Silencing of TNFSF10 inhibited the proliferation, migration and invasion of TGCTs cells, which is the first comprehensive investigation on the role of TNFSF10 in TGCTs. The expression of TNFSF10 could be a potential diagnostic and prognostic biomarker for patient stratification and drug therapy in patients with TGCTs.

## Data Availability

The datasets presented in this study can be found in online repositories. The names of the repository/repositories and accession number(s) can be found in the article/**Supplementary Material**.

## Supplementary material

Supplementary Figure 1 TNFSF10 expression profile across all tumor samples and paired normal tissues.

## References

[1] Soleimani M Kollmannsberger C Nappi L . Emerging role of biomarkers in testicular germ cell tumors. Curr Oncol Rep. (2022) 24:437–42. doi: 10.1007/s11912-022-01231-1. PMID: 35142973

[2] Tateo V Thompson ZJ Gilbert SM Cortessis VK Daneshmand S Masterson TA . Epidemiology and risk factors for testicular cancer: a systematic review. Eur Urol. (2024) 87:427–41. doi: 10.1016/j.eururo.2024.10.023. PMID: 39542769 PMC13098354

[3] Winter C Albers P . Testicular germ cell tumors: pathogenesis, diagnosis and treatment. Nat Rev Endocrinol. (2011) 7:43–53. doi: 10.1038/nrendo.2010.196. PMID: 21116298

[4] Cheng L Albers P Berney DM Feldman DR Daugaard G Gilligan T . Testicular cancer. Nat Rev Dis Primers. (2018) 4:29. doi: 10.1038/s41572-018-0029-0. PMID: 30291251

[5] Rajpert-De Meyts E McGlynn KA Okamoto K Jewett MAS Bokemeyer C . Testicular germ cell tumours. Lancet. (2016) 387:1762–74. doi: 10.1016/s0140-6736(15)00991-5. PMID: 26651223

[6] Yao X Zhou H Duan C Wu X Li B Liu H . Comprehensive characteristics of pathological subtypes in testicular germ cell tumor: gene expression, mutation and alternative splicing. Front Immunol. (2023) 13:1096494. doi: 10.3389/fimmu.2022.1096494. PMID: 36713456 PMC9883017

[7] Albers P Albrecht W Algaba F Bokemeyer C Cohn-Cedermark G Fizazi K . Guidelines on testicular cancer: 2015 update. Eur Urol. (2015) 68:1054–68. doi: 10.1016/j.eururo.2015.07.044. PMID: 26297604

[8] Chovanec M Lauritsen J Bandak M Oing C Kier GG Kreiberg M . Late adverse effects and quality of life in survivors of testicular germ cell tumour. Nat Rev Urol. (2021) 18:227–45. doi: 10.1038/s41585-021-00440-w. PMID: 33686290

[9] Mo L Yu Z Lv Y Cheng J Yan H Lu W . Single-cell RNA sequencing of metastatic testicular seminoma reveals the cellular and molecular characteristics of metastatic cell lineage. Front Oncol. (2022) 12:871489. doi: 10.3389/fonc.2022.871489. PMID: 35494058 PMC9039315

[10] Lv Z Hu J Su H Yu Q Lang Y Yang M . TRAIL induces podocyte PANoptosis via death receptor 5 in diabetic kidney disease. Kidney Int. (2024) 107:317–31. doi: 10.1016/j.kint.2024.10.026. PMID: 39571905

[11] ZhangH ChenY LiY ChenC WangB YinF . TNFSF10 drives hyperactive immune responses via NLRP3 inflammasome and endoplasmic reticulum stress in autoimmune and inflammatory diseases. J Adv Res. (2026), S2090-1232(26)00054-8. doi: 10.1016/j.jare.2026.01.029. PMID: 41548870

[12] Oh YT Sun SY . Regulation of cancer metastasis by TRAIL/death receptor signaling. Biomolecules. (2021) 11(4):499. doi: 10.3390/biom11040499. PMID: 33810241 PMC8065657

[13] Jiang W Wu D-B Fu S-Y Chen E-Q Tang H Zhou T-Y . Insight into the role of TRAIL in liver diseases. BioMed Pharmacother. (2019) 110:641–5. doi: 10.1016/j.biopha.2018.12.004. PMID: 30544063

[14] Wu C You M Nguyen D Wangpaichitr M Li Y-Y Feun LG . Enhancing the effect of tumor necrosis factor-related apoptosis-inducing ligand signaling and arginine deprivation in melanoma. Int J Mol Sci. (2021) 22(14):7628. doi: 10.3390/ijms22147628. PMID: 34299249 PMC8306073

[15] Becht E McInnes L Healy J Dutertre CA Kwok IWH Ng LG . Dimensionality reduction for visualizing single-cell data using UMAP. Nat Biotechnol. (2018). doi: 10.1038/nbt.4314. PMID: 30531897

[16] Liu Z Liu L Weng S Xu H Xing Z Ren Y . BEST: a web application for comprehensive biomarker exploration on large-scale data in solid tumors. J Big Data. (2023) 10:165. doi: 10.1186/s40537-023-00844-y, PMID: 38164791

[17] Tang Z Kang B Li C Chen T Zhang Z . GEPIA2: an enhanced web server for large-scale expression profiling and interactive analysis. Nucleic Acids Res. (2019) 47(W1):W556–W560. doi: 10.1093/nar/gkz430, PMID: 31114875 PMC6602440

[18] Cerami E Gao J Dogrusoz U Gross BE Sumer SO Aksoy BU . The cBio cancer genomics portal: an open platform for exploring multidimensional cancer genomics data. Cancer Discov. (2012) 2(5):401–4. doi: 10.1158/2159-8290.CD-12-0095, PMID: 22588877 PMC3956037

[19] Wang W Zhang J Wang Y Xu Y Zhang S . Identifies microtubule-binding protein CSPP1 as a novel cancer biomarker associated with ferroptosis and tumor microenvironment. Comput Struct Biotechnol J. (2022) 20:3322–35. doi: 10.1016/j.csbj.2022.06.046. PMID: 35832625 PMC9253833

[20] Peng D Wei J Gan Y Yang J Jiang X Kitazawa R . Testis developmental related gene 1 regulates the chemosensitivity of seminoma TCam-2 cells to cisplatin via autophagy. J Cell Mol Med. (2019) 23:7773–84. doi: 10.1111/jcmm.14654. PMID: 31496041 PMC6815826

[21] Gan Y Wang Y Tan Z Zhou J Kitazawa R Jiang X . TDRG1 regulates chemosensitivity of seminoma TCam-2 cells to cisplatin via PI3K/Akt/mTOR signaling pathway and mitochondria-mediated apoptotic pathway. Cancer Biol Ther. (2016) 17:741–50. doi: 10.1080/15384047.2016.1178425. PMID: 27104982 PMC4970539

[22] Guo J Grow EJ Mlcochova H Maher GJ Lindskog C Nie X . The adult human testis transcriptional cell atlas. Cell Res. (2018) 28:1141–57. doi: 10.1038/s41422-018-0099-2. PMID: 30315278 PMC6274646

[23] Bo H Zhu F Liu Z Deng Q Liu G Li R . Integrated analysis of high-throughput sequencing data reveals the key role of LINC00467 in the invasion and metastasis of testicular germ cell tumors. Cell Death Discov. (2021) 7:206. doi: 10.1038/s41420-021-00588-9. PMID: 34362879 PMC8346510

[24] Ganesh K Massagué J . Targeting metastatic cancer. Nat Med. (2021) 27:34–44. doi: 10.1038/s41591-020-01195-4. PMID: 33442008 PMC7895475

[25] Steeg PS . Targeting metastasis. Nat Rev Cancer. (2016) 16:201–18. doi: 10.1038/nrc.2016.25. PMID: 27009393 PMC7055530

[26] Mittal V . Epithelial mesenchymal transition in tumor metastasis. Annu Rev Pathol. (2018) 13:395–413. doi: 10.1146/annurev-pathol-020117-043854. PMID: 29414248

[27] Belli C Trapani D Viale G D'Amico P Duso BA Della Vigna P . Targeting the microenvironment in solid tumors. Cancer Treat Rev. (2018) 65:22–32. doi: 10.1016/j.ctrv.2018.02.004. PMID: 29502037

[28] Gao Y Yang J Cai Y Fu S Zhang N Fu X . IFN-γ-mediated inhibition of lung cancer correlates with PD-L1 expression and is regulated by PI3K-AKT signaling. Int J Cancer. (2018) 143:931–43. doi: 10.1002/ijc.31357. PMID: 29516506

[29] Han YJ Zhang J Hardeman A Liu M Karginova O Romero R . An enhancer variant associated with breast cancer susceptibility in Black women regulates TNFSF10 expression and antitumor immunity in triple-negative breast cancer. Hum Mol Genet. (2023) 32:139–50. doi: 10.1093/hmg/ddac168. PMID: 35930348 PMC9837834

[30] Zhang H Qin G Zhang C Yang H Liu J Hu H . TRAIL promotes epithelial-to-mesenchymal transition by inducing PD-L1 expression in esophageal squamous cell carcinomas. J Exp Clin Cancer Res. (2021) 40:209. doi: 10.1186/s13046-021-01972-0. PMID: 34167551 PMC8223376

[31] Chovanec M Mardiak J Mego M . Immune mechanisms and possible immune therapy in testicular germ cell tumours. Andrology. (2019) 7:479–86. doi: 10.1111/andr.12656. PMID: 31169364

[32] Schepisi G Gianni C Cursano MC Gallà V Menna C Casadei C . Immune checkpoint inhibitors and Chimeric Antigen Receptor (CAR)-T cell therapy: potential treatment options against testicular germ cell tumors. Front Immunol. (2023) 14:1118610. doi: 10.3389/fimmu.2023.1118610. PMID: 36860862 PMC9968831

