# Supplementary figures and images for: TNFSF10: a promising prognostic biomarker and therapeutic target for immunotherapy in testicular germ cell tumors
Source: Front Immunol. 2026 Apr 28;17:1761075. doi: 10.3389/fimmu.2026.1761075 (PMC13161098; doi:10.3389/fimmu.2026.1761075)

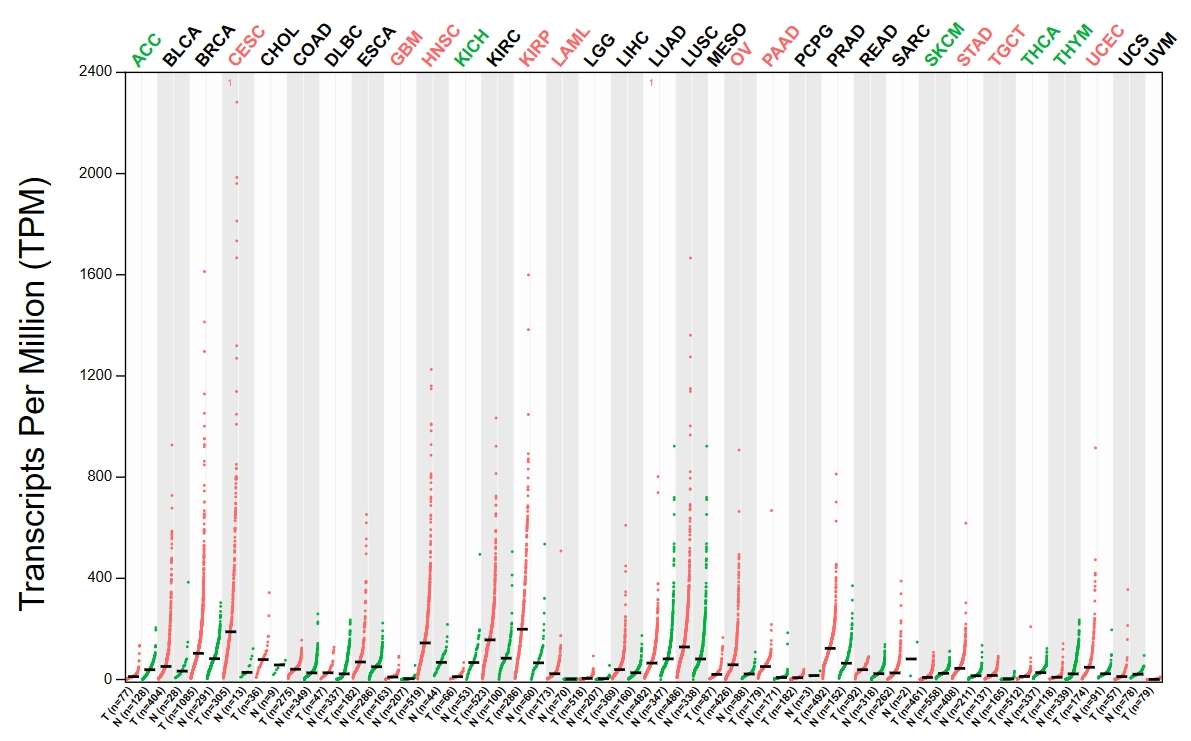


Supplement 1. TNFSF10 expression profile across all tumor samples and paired normal tissues.

Supplement: Supplementary Figure 1 — TNFSF10 expression profile across all tumor samples and paired normal tissues. [file DataSheet1.docx]
